# The Essential Role of Body Weight in Adjusting Gn Dosage to Prevent High Ovarian Response for Women With PCOS During IVF: A Retrospective Study

**DOI:** 10.3389/fendo.2022.922044

**Published:** 2022-07-01

**Authors:** Rujun Zeng, Hanxiao Chen, Xun Zeng, Lang Qin

**Affiliations:** ^1^Reproductive Centre, Department of Obstetrics and Gynaecology, West China Second University Hospital, Sichuan University, Chengdu, China; ^2^Key Laboratory of Birth Defects and Related Diseases of Women and Children of the Ministry of Education, West China Second University Hospital, Sichuan University, Chengdu, China

**Keywords:** polycystic ovarian syndrome, *in vitro* fertilization, body weight, gonadotropin, ovarian response

## Abstract

Polycystic ovarian syndrome (PCOS) is the major cause of anovulatory infertility. Since women with PCOS are often accompanied by increased body weight and hyper response to controlled ovarian stimulation, individualized gonadotropin (Gn) dose is required to achieve a therapeutic effect while minimizing the risk of ovarian hyperstimulation simultaneously. We aimed to investigate the essential role of body weight in optimizing initial Gn dosage for PCOS patients during *in vitro* fertilization (IVF). We retrospectively included 409 infertile PCOS patients who used gonadotropin-releasing hormone (GnRH)-antagonist fixed protocol and underwent their first cycle of IVF in West China Second University Hospital from January 2019 to June 2021. Baseline characteristics controlled ovarian stimulation parameters, and reproductive outcomes were compared between patients with different body weights and different ovarian responses. Multivariable linear regression analyses were adopted to investigate the relationship between body weight and initial Gn dosage. Receiver operating characteristic (ROC) curves were drawn to find the optimal cut-off value of body weight in predicting the starting Gn dosage so as to prevent high ovarian response (HOR). We found that luteinizing hormone (LH) level and Anti-Mullerian hormone (AMH) level were lowest in the group with body weight over 70 kg and was highest in the group with body weight less than 50 kg. Increased body weight was significantly correlated to the rise of initial Gn dosage (Beta = 0.399, t = 8.921, p < 0.001). Normal ovarian response (NOR) patients had significantly less fresh cycle cancel rate and ovarian hyperstimulation syndrome (OHSS) rate which outweighed the fewer embryos compared with HOR patients. Using ROC curves, 53.25 kg (sensitivity, 84.2%; specificity, 53.8%) and 70.5 kg (sensitivity, 58.8%; specificity, 93.0%) were identified as the optimal cut-off values to predict the initial Gn dosage of no more than 150 IU and 225 IU, respectively. In conclusion, adjusting the initial Gn dosage based on body weight is crucial to preventing ovarian hyperstimulation while not influencing reproductive outcomes for PCOS patients during IVF.

## Introduction

Polycystic ovarian syndrome (PCOS), a complex and multifaceted disorder characterized by hyperandrogenism, ovulatory dysfunction, and polycystic ovaries, has an increasing incidence rate of 4.47% and an age-standardized incidence rate of 1.45% per year globally (1). Approximately half of PCOS patients are overweight or obese and obesity plays an important role in the pathogenesis of PCOS and may aggravate the adverse metabolic outcomes of PCOS (2). About three-quarters of PCOS patients suffered from another common complication, infertility, making PCOS the major cause of anovulatory infertility (3). Assisted reproduction technology (ART), including *in vitro* fertilization (IVF) and embryo transfer (ET) and intracytoplasmic sperm injection (ICSI), is often needed when patients are resistant to ovarian induction or complicated with other infertility factors.

Women with PCOS exhibit higher sensibility and exaggerated response to gonadotropins which could result in an increased risk of ovarian hyperstimulation. Ovarian hyperstimulation syndrome (OHSS) is a serious iatrogenic complication characterized by fluid shifting from intravascular to extravascular spaces due to arteriolar vasodilatation and increased capillary permeability (4). It occurs as mild type in 20%-30% of IVF cycles and may develop to moderate or severe type in 2%-3% of cycles (5). Individualized exogenous gonadotropin (Gn) dose is essential for minimizing OHSS risk and optimizing follicle recruitment at the same time. The strategies to manage OHSS include initial dosage selection and dose adjustment during cycle (6). Several studies have developed a series of algorithms to predict the proper initial dosage of Gn based on age, Anti-Mullerian hormone (AMH), body mass index (BMI), baseline follicle stimulating hormone (FSH) level, or ovarian response of the previous cycle (7). Obesity also takes an essential part in ovarian response and could augment adverse reproductive outcomes (2). However, these studies were lacking in evidence-based on body weight and regimens aimed at PCOS patients were limited.

Body weight is one of the factors associated with pharmacokinetic parameters. Patients with different body wight require dose adjustment to achieve equivalent therapeutic effects. Some had reported that body weight is more important than BMI in determining the dosage of exogenous Gn (8). Studies have found that body weight was negatively associated with exogenous Gn levels (8). One study reported that weight-adjusted rFSH dose could predict follicular growth and retrieval in general women, while the complications and pregnancy outcomes were not reported (9). As a large number of PCOS patients are accompanied by increased body weight, upregulating Gn dose is often needed to achieve the therapeutic effect, however, this in turn may increase the risk of ovarian hyperstimulation. Therefore, it is important to weigh the pros and cons and find the balance when adjusting Gn dosage according to body weight.

In this retrospective cohort study, we aimed to investigate the association between body weight and individualized Gn dosage and the ART outcomes in women with PCOS undergoing IVF cycles. In addition, we tried to find the optimal cut-off value of body weight in predicting the increase of initial Gn dosage in PCOS patients in order to prevent high ovarian response (HOR).

## Materials and Methods

### Study Population

In this study, we retrospectively enrolled infertile patients diagnosed with PCOS who underwent their first cycle of IVF in the Reproductive Center, Department of obstetrics and gynecology, West China Second University Hospital from January 2019 to June 2021. The Ethical Review Board of West China Second University Hospital, Sichuan University, approved the study and waived the need for written informed consent (Approval No. 2021-033).

PCOS was diagnosed according to the European Society for Human Reproduction and Embryology/American Society for Reproductive Medicine (ESHRE/ASRM) (Rotterdam criteria) (10). Only the first controlled ovarian stimulation (COS) cycles treated with gonadotropin-releasing hormone (GnRH)-antagonist fixed protocol were included. Exclusion criteria include: male factor infertility, other COS protocols (e.g., GnRH-antagonist flexible protocol, depot GnRH-agonist protocol, long GnRH-agonist protocol, etc.); second or further COS cycles; female with any known systemic or endocrine diseases, such as Cushing syndrome, androgen secreting ovarian tumors or adrenal tumors, functional hypothalamic amenorrhea (FHA), thyroid diseases, hyperprolactinemia, premature ovarian insufficiency (POI); couples with abnormal chromosome karyotype not including chromosome polymorphisms; female with a history of recurrent spontaneous abortion.

Baseline clinical characteristics including age, type of infertility, oligo/hypomenorrhea, height, weight, and BMI were extracted from the patient records. Laboratory examination data including baseline testosterone (T) level, dehydroepiandrosterone sulfate (DHEAS) level, androstadienone (AND) level, sex hormone-binding globulin (SHBG) level, FSH level, luteinizing hormone (LH) level, polycystic ovarian morphology (PCOM) measured by antral follicle count (AFC) level, and AMH level were also collected. Sex hormones and ultrasonographic examinations were measured on day 2-4 of the menstrual cycle.

### Controlled Ovarian Stimulation Protocols

As mentioned above, only patients who were treated with fixed GnRH-antagonist protocol were included in this study. Briefly, exogenous Gn (Gonal-F, Merck Serono, Germany), generally 100-375 IU/day, was administered starting from day 2-3 of menstruation. The doses were determined by experienced (more than 10 years) fertility/infertility specialists based on the patient’s age, BMI, PCOM, and follicular response. GnRH-antagonist (Cetrotide, 0.25 mg, Merck Serono, Germany) was daily administrated after 5-7 days usage of Gn and the administration of GnRH-antagonist continued until triggering. Urine human chorionic gonadotropin (hCG) (Ovidrel, 8000-10000 U, Merck-Serono, Germany) was given to trigger ovulation when two leading follicles reached a mean diameter of 18 mm, or three follicles reached a mean diameter of 17 mm. A decreased dose of urine hCG (5000 IU), recombinant hCG (250 ug), or GnRH agonist (0.2 mg) with urine hCG (2000 IU) was used to trigger ovulation when patients were at high risk of OHSS. Initial Gn dosage, stimulation time, total Gn dosage, number of oocytes with diameter ≥14 mm on trigger day, estradiol (E2), progesterone (P), LH, endometrial thickness on trigger day measured by sonographic examinations, and number of oocytes retrieved were recorded.

### Fresh Embryo Transfer Cycle

Oocytes were retrieved transvaginally 36-38 h after the trigger. If the patients showed increased progesterone level or at high risk of OHSS, fresh ET cancellation and freeze-all strategy were applied. All the additional embryos were cryopreserved. The morphology of embryo or blastocyst was assessed to determine its quality (11). The day 3 embryo was defined as good-quality if it presented two pronuclei (PN) when fertilization, had six to 10 blastomeres and no more than 20% fragmentation. The blastocyst was defined as good-quality if it met with the inner cell mass/trophectoderm score of AA, AB, BA, or BB.

In the present study, data including mature (MII) oocyte rate, IVF fertilization rate, IVF normal fertilization rate, ICSI fertilization rate, ICSI normal fertilization rate, cleavage rate, good-quality D3 embryo rate, blastocyst formation rate, good-quality blastocyst rate, fresh ET cancellation rate, severe OHSS rate, clinical pregnancy rate after fresh ET, and cumulative clinical pregnancy rate were collected. Fertilization was defined as the presence of PN 16-18 h post-insemination or post-injection. Normal fertilization was defined as the presence of 2PN on D1 post-insemination or post-injection. In this study, HOR was defined as a patient who had at least one of the following features (12) (1): >15 retrieved oocytes during COS cycle or cycle cancellation due to excessive follicular development (2); > 20 oocytes larger than 12-14mm in diameter during COS cycle (3); moderate or severe OHSS after COS. Poor ovarian response (POR) was defined as patients who had at least two of the following three characteristics (13) (1): maternal age ≥40 years or any other known risk factor for POR (2); previous POR history (i.e., retrieved less than three oocytes under a conventional stimulation protocol) (3); abnormal ovarian reserve test. And the rest was defined as the normal ovarian response (NOR). Serum hCG test was conducted 14 days after ET and transvaginal ultrasound (TVS) was done 28 days after ET. Clinical pregnancy was recorded when the gestational sac was observed by TVS. Patients who were hospitalized because of severe OHSS were recorded.

### Statistical Analyses

We first divided the participants into four groups based on body weight: body weight between 40-50 kg (Group A), body weight between 50-60 kg (Group B), body weight between 60-70 kg (Group C), and body weight greater than 70 kg (Group D). Continuous variables were expressed as mean ± standard deviation (SD) and category variables were displayed as frequency (n) and percentage (%). One-way analysis of variance (ANOVA) test was applied to compare normal distribution continuous variables between the four groups and LSD test was used as the *post hoc* test, and median and interquadrant range (IQR) and Kruskal-Wallis H tests were used to compare abnormal distribution variables. Chi-square test and/or Fisher’s exact test were used for the comparison between the four groups as appropriate, and Bonferroni correction was used for comparison between every two groups. Multivariable linear regression analyses were also performed to compare the set-up of initial Gn dosage. Age, weight, T, LH, and AMH were used in the regression model. Afterward, we compared the reproductive outcomes between PCOS patients with HOR and NOR. While the student’s t-test was adopted for the comparison between two normal distributed continuous variables, Mann-Whitney U test was used to compare two abnormal distributed continuous variables, Chi-square test and/or Fisher’s exact test were used to compare category variables between the aforementioned two groups. A receiver operating characteristic (ROC) curve was used to identify a cut-off value for body weight to accurately predict the need of increasing initial Gn dosage. The optimized cut-off value was selected where the ROC curve reached the maximum area under the curve (AUC) with the greatest sum of sensitivity and specificity. For all comparisons, a two-sided p-value less than 0.05 was considered statistically significant. All statistical analyses were performed using SPSS version 22.0 (IBM, Armonk, NY, USA).

## Results

### Clinical Characteristics and Reproductive Outcomes Between PCOS Patients of Different Body Weight Groups

A total of 409 women diagnosed with PCOS who used GnRH-antagonist fixed protocol as COS protocol were included in the present study. Among the four groups, the group with body weight over 70 kg had the lowest LH and AMH level while the group with body weight less than 50 kg had the highest LH and AMH level. Except for BMI, other baseline characteristics were similar among the four groups (all p > 0.05). Details of baseline clinical characteristics of the PCOS participants are shown in **Table 1**.

**Table 1:** Baseline characteristics of PCOS patients in four body weight groups (n = 409).

Characteristics		Weight 40~50kg Group	Weight 50~60 kg Group	Weight 60~70 kg Group	Weight ≥70 kg Group	P
		(n = 63)	(n = 188)	(n = 109)	(n = 49)	
Age (years)		28 (26-30.5)	29 (27-32)	29 (26.3-31)	29 (26-31)	0.513
Type of infertility	Primary	68.25% (43/63)	63.83% (120/188)	70.64% (77/109)	51.02% (25/49)	0.106
	Secondary	31.75% (20/63)	36.27% (68/188)	29.36% (32/109)	48.98% (24/49)
Oligo/hypomenorrhea		90.48% (57/63)	84.57% (159/188)	88.99%(97/109)	83.67% (41/49)	0.499
BMI (kg/m2)		18.8 (17.3-19.7)	21.4 (20.3-22.6)^ab^	24.5 (23.4-25.9)^cd^	28.2 (27.3-28.9)^ef^	<0.001*
T (ng/ml)		0.5 (0.3-0.6)	0.4 (0.3-0.5)	0.4 (0.3-0.6)	0.4 (0.3-0.8)	0.266
DHEAS (ug/dl)		0 (0-186)	90.1 (0-204.8)	0 (0-235.3)	135 (0-221.5)	0.725
AND (ng/ml)		0 (0-3.2)	1.7 (0-3.3)	0 (0-3.1)	1.3 (0-4.2)	0.604
SHBG (nmol/L)		0 (0-56)	0 (0-40.7)	0 (0-21.4)	6.8 (0-22.9)	0.237
FSH		6.6 (5.9-7.9)	6.6 (5.7-8.1)	6.3 (5.6-7.4)	6.7 (5.9-7.5)	0.646
LH		12.6 (8.3-17.9)	8.6 (5.6-14.7)	7.9 (4.7-11)^c^	6.1 (3.9-11.2)^e^	0.014*
LH/FSH		2.1 (1.1-2.9)	1.3 (0.8-2.2)	1.3 (0.8-1.8)	1 (0.6-1.8)	0.031
PCOM		80.95% (51/63)	79.26% (149/188)	84.40% (92/109)	83.67% (41/49)	0.706
AMH (ng/ml)		12.4 (8.7-15.5)	10.5 (6.9-15.7)	9.7 (5.8-13.3)	4.8 (3.1-8.3)^ef^	<0.001*

BMI, body mass index; T, testosterone; DHEAS, dehydroepiandrosterone sulfate; AND, androstadienone; SHBG, sex hormone-binding globulin; FSH, follicle-stimulating hormone; LH, luteinizing hormone; PCOM, polycystic ovarian morphology; AMH, Anti-Mullerian hormone. *, P values < 0.05; ^a^p<0.05 between Group A and Group B; ^b^p<0.05 between Group B and Group C; ^c^p<0.05 between Group A and Group C; ^d^p<0.05 between Group C and Group D; ^e^p<0.05 between Group A and Group D; ^f^p<0.05 between Group B and Group D.

Initial Gn dosage and total dosage of Gn were significantly higher in the group with higher body weight (Group A = 150 (125-150), Group B = 150 (150-184.4), Group C = 175 (150-225), Group D = 212.5 (175-300), p < 0.001, and Group A = 1350 (1125-1512.5), Group B = 1575 (1350-1846.9), Group C = 1775 (1500-2250), Group D = 2137.5 (1800-2587.5), p < 0.001, respectively). Notably, more patients presented HOR in the group with body weight less than 50 kg, with 65.08% HOR patients in Group A, 64.89% in Group B, 60.55% in Group C, and 40.82% in Group D (p = 0.018). The clinical outcomes between the four weight groups were similar, except for blastocyst formation rate and good-quality blastocyst rate (76.71% vs. 73.17% vs. 72.60% vs. 63.94%, p = 0.003 and 27.21% vs. 35.40% vs. 29.66% vs. 35.95%, p = 0.018, respectively). Details of the COS and clinical outcomes of the four weight groups are displayed in **Table 2**.

**Table 2:** COS and clinical outcomes of PCOS patients in four body weight groups (n = 409).

Characteristics		Weight 40~50kg Group	Weight 50~60 kg Group	Weight 60~70 kg Group	Weight ≥70 kg Group	P
		(n = 63)	(n =188)	(n = 109)	(n = 49)	
Initial Gn dosage (IU/day)		150 (125-150)	150 (150-184.4)^ab^	175 (150-225)^cd^	212.5 (175-300)^ef^	<0.001*
Stimulation time (days)		9 (8.5-10)	10 (9-11)	10 (9.3-11)^c^	10 (9-12)^e^	<0.001*
Total dosage of Gn (IU)		1350 (1125-1512.5)	1575 (1350-1846.9)^ab^	1775 (1500-2250)^cd^	2137.5 (1800-2587.5)^ef^	<0.001*
Number of oocytes with diameter ≥14mm on hCG day		12 (8-13.5)	10 (7-13)	9 (8-11.8)^c^	8 (7-9.3)^e^	0.009*
E2 on hCG day (pg/ml)		5200.3 (3470.2-8891.9)	5128.2 (3249.8-7094)	4254.8 (2776.2-6748.7)	2786.5 (1989-3793.9)^ef^	<0.001*
P on hCG day (ng/ml)		1.2 (0.8-1.4)	1 (0.8-1.5)	0.9 (0.6-1.3)	0.7 (0.5-1.1)^ef^	0.009*
LH on hCG day (IU/L)		1.7 (1.1-3.1)	1.7 (0.9-3.5)	2.1 (1.1-3.2)	2.4 (1.2-3.1)^e^	0.012*
Endometrial thickness on hCG day (mm)		5 (4.5-5.6)	5 (4.2-5.6)	5 (4.4-5.5)	5 (4.2-5.4)	0.283
Ovarian response	High	65.08% (41/63)	64.89% (122/188)	60.55% (66/109)	40.82% (20/49)^gh^	0.018*
	Normal	34.92% (22/63)	35.11% (66/188)	39.45% (43/109)	59.18% (29/49)
Number of oocytes retrieved		13 (9.5-20)	16 (11-21.8)	15 (10-20)	12 (9.8-16.3)	0.06
MII oocyte rate		83.66% (814/973)	84.52% (2729/3229)	84.78% (1521/1794)	86.69% (573/661)	0.405
IVF fertilization rate		74.86% (658/879)	74.14% (2340/3156)	72.73% (1264/1738)	78.23% (485/620)	0.06
IVF normal fertilization rate		60.87% (574/943)	60.15% (1923/3197)	58.94% (1035/1756)	58.85% (389/661)	0.706
ICSI fertilization rate		85.95% (104/121)	91.60% (218/238)	87.10% (162/186)	88.68% (47/53)	0.334
ICSI normal fertilization rate		79.34% (96/121)	87.17% (197/226)	79.03% (147/186)	81.13% (43/53)	0.119
Cleavage rate		97.64% (744/762)	98.28% (2514/2558)	98.60% (1406/1426)	98.50% (524/532)	0.411
Good-quality D3 embryo rate		55.96% (310/554)	52.54% (952/1812)	51.15% (512/1001)	53.74% (201/374)	0.322
Blastocyst formation rate		76.71% (326/425)	73.17% (982/1342)	72.60% (583/803) ^i^	63.94% (172/269)^gh^	0.003*
Good-quality blastocyst rate		27.21% (83/305)	35.40% (314/887)^j^	29.66% (159/536)	35.95% (55/153)^g^	0.018*
Fresh ET cancellation rate		76.19% (48/63)	66.49% (125/188)	70.64% (77/109)	53.06% (26/49)	0.06
Severe OHSS rate		4.76% (3/63)	3.19% (6/188)	1.83% (2/109)	2.04% (1/49)	0.753
Clinical pregnancy rate after fresh ET		66.67% (10/15)	50.79% (32/63)	50.00% (16/32)	52.17% (12/23)	0.715
Cumulative clinical pregnancy rate		64.10% (25/39)	53.85% (70/130)	57.58% (38/66)	60.00% (18/30)	0.694

Gn, gonadotrophin; E2, estradiol; P, progesterone; LH, luteinizing hormone; OSI, ovarian sensitivity index; MII, mature; IVF, in vitro fertilization; ICSI, intracytoplasmic sperm injection; ET, embryo transfer; OHSS, ovarian hyperstimulation syndrome. *P values < 0.05; ^a^p<0.05 between Group A and Group B; ^b^p < 0.05 between Group B and Group C; ^c^p < 0.05 between Group A and Group C; ^d^p < 0.05 between Group C and Group D; ^e^p < 0.05 between Group A and Group D; ^f^p < 0.05 between Group B and Group D; ^g^p < 0.0125 between Group A and Group D; ^h^p < 0.0125 between Group B and Group D; ^i^p < 0.0125 between Group C and Group D; ^j^p < 0.0125 between Group A and Group B.

### Clinical Characteristics and Reproductive Outcomes Between Different Ovarian Response Groups

There were 249 patients of HOR and 160 patients of NOR in our study. And none of our patients expressed POR. Compared with PCOS patients with NOR, those with HOR had lower body weight, higher AMH level, higher LH to FSH ratio, and less percentage of PCOM. During COS, lower initial Gn dosage (150 (150-200) vs. 175 (150-225), p = 0.008), lower total Gn dosage (1575 (1275-1850) vs. 1725 (1425-2193.8), p = 0.007), and more oocytes retrieved (19.5 (16-24) vs. 10 (8–12), p < 0.001) were observed in HOR patients. Patients with HOR had higher MII oocyte rate (85.26% vs. 82.77%, p = 0.017) and IVF normal fertilization rate (60.45% vs. 57.65%, p = 0.050), lower good-quality D3 embryo rate (51.27% vs. 58.55%, p < 0.001) than the NOR. Although clinical pregnancy rate after fresh ET and cumulative clinical pregnancy rate did not differ between patients with HOR and those with NOR, HOR patients had significantly higher fresh ET cancellation rate and severe OHSS rate (89.96% vs. 32.50%, p < 0.001 and 4.42% vs. 0.63%, p = 0.033, respectively). Details of the comparison of HOR and NOR PCOS patients are displayed in **Table 3**.

**Table 3:** Comparison of characteristics in PCOS patients with high ovarian response and normal ovarian response (n = 409).

Characteristics		HOR group	NOR group	P
		(n = 249)	(n = 160)	
Age (years)		29 (27-32)	29 (27-31)	0.722
Type of infertility	Primary	66.67% (166/249)	61.88% (99/160)	0.322
	Secondary	33.33% (83/249)	38.12% (61/160)
Oligo/hypomenorrhea (%)		87.95% (219/249)	84.38% (135/160)	0.301
Weight (kg)		55 (51-60)	57 (52-65)	0.015*
BMI (kg/m2)		21.8 (20.3-23.6)	22.9 (20.3-25.6)	0.041*
T (ng/ml)		0.4 (0.3-0.5)	0.4 (0.3-0.6)	0.036*
DHEAS (ug/ml)		99.3 (0-210.5)	0 (0-204.5)	0.831
AND (ng/ml)		1.8 (0-3.4)	0 (0-3)	0.462
SHBG (nmol/l)		0 (0-41.9)	0 (0-22.6)	0.064*
FSH		6.5 (5.6-7.6)	6.5 (5.9-8)	0.022*
LH		9.6 (6.4-14.8)	7 (4.5-11.2)	0.001*
LH/FSH		1.6 (1-2.4)	1.1 (0.7-1.7)	<0.001*
AMH (ng/ml)		12 (9-16)	6.9 (4.1-11.6)	<0.001*
PCOM		85.94% (214/249)	74.38% (119/160)	0.003*
Initial Gn dosage (IU/day)		150 (150-200)	175 (150-225)	0.008*
Stimulation time (days)		10 (9-11)	10 (9-11)	0.467
Total Gn dosage (IU)		1575 (1275-1850)	1725 (1425-2193.8)	0.007*
Number of oocytes retrieved		19.5 (16-24)	10 (8-12)	<0.001*
MII oocyte rate		85.26% (4350/5102)	82.77% (1287/1555)	0.017*
IVF fertilization rate		74.34% (3644/4902)	73.98% (1103/1491)	0.781
IVF normal fertilization rate		60.45% (3039/5027)	57.65% (882/1530)	0.050*
ICSI fertilization rate		89.94% (438/487)	83.78% (93/111)	0.064
ICSI normal fertilization rate		83.37% (406/487)	77.78% (77/99)	0.183
Cleavage rate		98.26% (4011/4082)	98.41% (1177/1196)	0.723
Good-quality D3 embryo rate		51.27% (1516/2957)	58.55% (459/784)	<0.001*
Blastocyst formation rate		72.88% (1701/2334)	71.68% (362/505)	0.585
Good-quality blastocyst rate		32.89% (523/1590)	30.24% (88/291)	0.374
Fresh ET cancellation rate		89.96% (224/249)	32.50% (52/160)	<0.001*
Severe OHSS rate		4.42% (11/249)	0.63% (1/160)	0.033*
Clinical pregnancy rate after fresh ET		56.00% (14/25)	51.85% (56/108)	0.708
Cumulative clinical pregnancy rate		59.71% (83/139)	53.97% (68/126)	0.346

HOR, high ovarian response; NOR, normal ovarian response; BMI, body mass index; T, testosterone; DHEAS, dehydroepiandrosterone sulfate; AND, androstadienone; SHBG, sex hormone-binding globulin; FAI, free androgen index; FSH, follicle-stimulating hormone; LH, luteinizing hormone; PCOM, polycystic ovarian morphology; AMH, Anti-Mullerian hormone; Gn, gonadotrophin; E2, estradiol; P, progesterone; OSI, ovarian sensitivity index; MII, mature; IVF, in vitro fertilization; ICSI, intracytoplasmic sperm injection; ET, embryo transfer; OHSS, ovarian hyperstimulation syndrome. *P values < 0.05.

### The Role of Body Weight in Adjusting Gn Dosage to Prevent HOR

In our multiple linear regression analysis, we aimed to find the association between body weight and initial Gn dosage, and the model was adjusted for age, body weight, T, LH, and AMH. We found that increased body weight was an independent factor that was significantly associated with the increase in initial Gn dosage (Beta = 0.399, t = 8.921, p < 0.001). In addition, AMH was inversely correlated with initial Gn dosage (Beta = -0.246, t = -5.458, p < 0.001). Patient’s age, T, and LH level were not associated with initial Gn dosage in our analysis (**Table 4**).

**Table 4:** Multiple linear regression analysis of initial Gn dosage.

	Unstandardized coefficients		Standardized coefficients	t	P
	B	SE	Beta		
Age	-0.56	0.698	-0.035	-0.802	0.423
Weight	2.579	0.289	0.399	8.921	<0.001
T	1.756	4.24	0.018	0.414	0.679
LH	0.059	0.205	0.013	0.287	0.774
AMH	-2.87	0.526	-0.246	-5.458	<0.001

SE, standard error; T, testosterone; LH, luteinizing hormone; AMH, Anti-Mullerian hormone.

All patients presenting with NOR received an initial Gn dosage between 100 IU and 300 IU. A ROC curve was drawn to identify the optimal cut-off value of body weight in predicting the increase of initial Gn dosage of over 150 IU and 225 IU. As for initial Gn dose of over 150 IU, the AUC was 0.725 (95% CI: 0.645-0.805, p < 0.001). Body weight of 53.25 kg was selected as the optimal cut-off value with a sensitivity of 84.2% and a specificity of 53.8% (**Figure 1A**). The AUC was 0.843 (95% CI: 0.755-0.932, p < 0.001) for initial Gn dose of over 225 IU, and the cut-off value was 70.5 kg for body weight (sensitivity, 58.8%; specificity, 93.0%) (**Figure 1B**).

**Figure:**
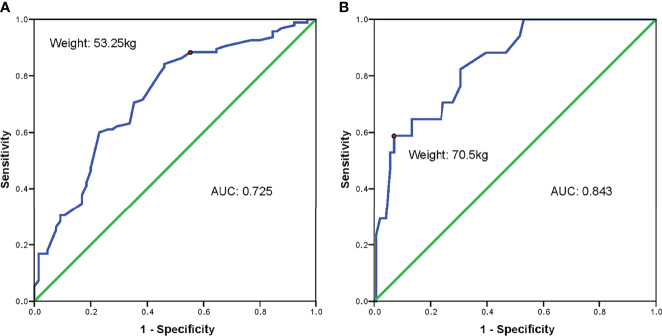
**Figure 1** Receiver operating characteristic curves of body weight in predicting initial Gn dosage set up in PCOS patients with normal ovarian response. **(A)** body weight in predicting initial Gn dosage higher than 150 IU/day in PCOS patients with normal ovarian response; **(B)** body weight in predicting initial Gn dosage higher than 225 IU/day in PCOS patients with normal ovarian response. AUC, area under curve.

## Discussion

In this retrospective cohort study, we found that increased body weight was significantly correlated to the increase of initial Gn dosage which was also associated with HOR. Compared with HOR patients, NOR patients had significantly less fresh ET cancellation rate and severe OHSS rate. In this way, we believed that adjusting initial Gn dosage based on body weight may be beneficial to reproductive outcomes in PCOS patients. Therefore, using ROC curves, we found that less than 150 IU initial Gn was appropriate for patients with body weight under 53.25 kg, and less than 225 IU initial Gn was suitable for those under 70.5 kg in order to prevent HOR.

According to our results, we found that there was a relationship between initial Gn dose and body weight and AMH. An appropriate dosage of Gn is necessary during ovarian stimulation to improve synchronization of follicular growth and maturity of oocytes at retrieval and avoid unpredicted POR at the same time (14). For young women with AFC>15, rFSH dose (IU per kg) was related to ovarian response, and the starting dose of rFSH adjusted for body weight had a prediction role on day 5 median follicle size and the proportion of antral follicles recruited, when adjusted by age, AFC, and pre-treatment FSH level (9). Several factors have been put forward to adjust the Gn dose during COS, including ovarian response, AMH, and AFC (6, 15, 16). Involving two or more factors could help improve COS results significantly. Some had individualized rFSH doses based on the consistency in r-FSH starting doses for individualized treatment (CONSORT) dosing algorithm (17). However, patients with more than 25 oocytes retrieved, a history of severe OHSS, or BMI over 30 kg/m^2^ were excluded. One study set the optimal number of oocytes retrieval to be 9 in women younger than 40 but they also failed to include women with irregular menstrual cycle or presented PCOM (18). Another two PIVET rFSH algorithms adjusted by AMH, AFC, BMI, and age were conducted to optimize the number of retrieved oocytes of no more than 15 (19). The researchers found that the need for elevating rFSH dose increased with the starting dose reduction. The cancellation rate of all and no more than 75 IU FSH groups were 6.2% and 8.7%, respectively. Unfortunately, the clinical pregnancy and live birth outcomes of hyper-responders were not provided.

A higher incidence of HOR in patients with relatively lower body weight was observed in our study, although they received less initial and total Gn dose during COS. Compared with those who showed NOR, patients with HOR, although retrieved more oocytes, had about eight times the incidence of OHSS and 2.5 times of fresh ET cancellation rate. Limited studies have focused on the ovarian stimulation of hyper-responders. A meta-analysis summarized the effect of Gn dose grouped by ovarian reserve tests (20). Only two studies were included in predicted hyper-responders’ part (AFC>15 or AMH 15-50 pmol/l). The results showed that decreasing the dose from 150IU did not make a difference in clinical pregnancy and live birth rates, while it did reduce the risk of moderate and severe OHSS. Similar results were reported by a previous study that lowered the FSH dose in patients with AMH >32 pmol/L (21). Our results together with these findings showed that the weakening of actual ovarian response reflected by oocytes retrieval and transferable embryos did not outweigh clinical outcomes. As the clinical outcomes would not be improved or even impaired when more than 15-20 oocytes were recovered, together with increased risk of early OHSS or thromboembolic events (22). It is reasonable and vital to control the incidence of HOR. We also found that HOR women expressed relatively higher AMH, LH to FSH ratio, and PCOM, indicating these patients had more serious endocrine dysfunction. AMH and AFC are important indicators of Gn dose. Through regression analysis, we found that body weight is also an independent factor of the starting dose. Since AMH and AFC are generally high in PCOS patients, it is necessary to take body weight into consideration.

According to ESHRE guideline recommendations, a Gn dose of 150 IU in GnRH antagonist protocol was suggested for high responders (23). However, the pharmacokinetics and pharmacodynamics of Gn should be individualized to different patients. The excess weight affects the ovarian response to Gn and exogenous serum FSH level is inversely associated with body weight (24). The volume of extracellular fluid is a key factor for drug distribution. Women with elevated BMI own a larger portion of fat tissue, which contributes to low content of extracellular water than those without (8). In other words, for two patients with the same BMI, the one with a higher body weight owns more extracellular fluid than the other. Therefore, body weight is more predominant in determining FSH distribution than BMI (25). A previous study found that reducing the FSH dose in predicted hyper responders with body weight > 55kg significantly decreased the OHSS occurrence but also decreased the probability of live birth (24). On the contrary, a randomized controlled trial (RCT) found that the reduction of Gn dosage significantly lowered the OHSS incidence, together with no influence on live birth rates (11). However, women involved in this study were free from PCOS. Some researchers came up with a low-dose stimulation with a basement dose of 75 IU and increment/decrement of 25-50 IU according to age, AMH, BMI, and previous onset of OHSS. Patients with PCOS and the control group had comparable clinical pregnancy rates (32.2% vs. 34.4%) and moderated or severe OHSS rates (16.9% vs. 15.7%). Additionally, there was no cancellation because of unexpected poor responses (7). One study made a cut-off value of 60 kg to decrease FSH dose from 150 IU to 112.5 IU in PCOS patients but did not explain the possible reason behind it (26). In our study, as all patients presented HOR or NOR, no patients received a starting dose of over 300 IU as Gn overdose did not lead to increased oocytes and pregnancy outcomes (27). We aimed to clarify the exact body weight to predict the need for lowering the initial Gn dosage from 150 IU or 300 IU, to prevent the risk of HOR. We suggested that patients with body weight below 53.25 kg required a starting dose of Gn of less than 150 IU, and 70.5 kg for Gn dose less than 225 IU, based on the data from NOR patients.

However, our study also has several limitations. Firstly, as a retrospective study, there might be selective bias. Therefore, we collected and compared the baseline characteristics of the enrolled participants. Secondly, the sample size of our study was relatively small, which might cause the insignificance of some findings due to limited power. Thirdly, this study was conducted in a single reproductive center which may limit the external validity of our findings. Also, we were not able to access the pregnancy results and cumulative live birth rate due to the limited following-up time. Further multicenter studies with larger sample sizes and a longer follow-up period are needed.

In summary, our result showed a relationship between body weight and Gn starting dose in ART, which was often overlooked by previous studies. Adjusting initial Gn dosage according to body weight is of great importance for preventing HOR and better reproductive outcomes in PCOS patients and further large scale, randomized, controlled trials should be encouraged in this field.

## Data Availability Statement

The raw data supporting the conclusions of this article will be made available by the authors, without undue reservation.

## Ethics Statement

The studies involving human participants were reviewed and approved by The Ethical Review Board of West China Second University Hospital, Sichuan University. The ethics committee waived the requirement of written informed consent for participation.

## Author Contributions

XZ and LQ designed the study. RZ had full access to all the data in the study. HC performed the statistical analyses. RZ, HC, XZ, and LQ contributed to the clinical interpretation of the results. RZ and HC drafted the manuscript. All authors read and approved the final version of the article.

## Funding

This work was supported by the Sichuan Science and Technology Program [2020YFS0127].

## Conflict of Interest

The authors declare that the research was conducted in the absence of any commercial or financial relationships that could be construed as a potential conflict of interest.

## Publisher’s Note

All claims expressed in this article are solely those of the authors and do not necessarily represent those of their affiliated organizations, or those of the publisher, the editors and the reviewers. Any product that may be evaluated in this article, or claim that may be made by its manufacturer, is not guaranteed or endorsed by the publisher.
